# Sexual minority status, school-based violence, and current tobacco use among youth

**DOI:** 10.18332/tpc/156110

**Published:** 2022-12-15

**Authors:** Cherdsak Duangchan, Alicia K. Matthews, Ariel U. Smith, Alana D. Steffen

**Affiliations:** 1 College of Nursing, University of Illinois at Chicago, Chicago, United States; 2 Faculty of Nursing, HRH Princess Chulabhorn College of Medical Science, Chulabhorn Royal Academy, Bangkok, Thailand

**Keywords:** sexual and gender minorities, LGBTQ, school-based violence, tobacco use, youth

## Abstract

**INTRODUCTION:**

Sexual minority individuals experience elevated risk for smoking and violence due to a combination of general and unique identity-based risk factors. This study examined associations among sexual minority status, school-based violence, and tobacco use, among youth.

**METHODS:**

Data for this secondary data analysis consisted of Chicago-specific data from the 2019 Youth Risk Behavior Surveillance System (n=1562). Current use (≥1 day during the previous 30 days) of any tobacco product (cigarettes, e-cigarettes, smokeless tobacco, and cigars) and school-based violence (avoided school because they felt unsafe, were threatened/injured with a weapon, were in a physical fight, and were bullied) were estimated by sexual orientation (heterosexual vs gay, lesbian, bisexual, and unsure). A chi-squared test was used to investigate associations among the variables. Path analysis was employed to examine possible mediation effects of school-based violence.

**RESULTS:**

Thirty percent of sexual minority youth and 11.5% of heterosexual youth reported current tobacco use (χ^2^=55.91; p<0.001). Nearly one-third (31.8%) of youth reported school-based violence, with a higher rate (41.2%) reported by sexual minority youth compared to heterosexual youth (28.1%; χ^2^=19.48; p<0.001). Path analysis confirmed these associations, controlling for sex, age, and race/ethnicity. The model showed that sexual minority status increased odds of current tobacco use by a factor of 1.8 (95% CI: 1.3–2.6) via its relationship with school-based violence, explaining 33.8% of the total association between sexual minority status and tobacco use.

**CONCLUSIONS:**

Tobacco use was higher among sexual minority youth. School-based violence partially mediated the association between sexual minority status and tobacco use. Findings highlight the need for tobacco prevention and treatment efforts for sexual minority youth and school-based interventions to reduce exposure to violence.

## INTRODUCTION

Tobacco use is the leading cause of preventable disease and death in the US^1^. More than 80% of all adult smokers initiate tobacco use prior to the age of 18 years^2^. As such, tobacco use by youth presents a significant public health concern. Over the past 25 years, considerable progress has been made in reducing combustible-cigarette smoking among high school students^3^. However, nicotine-containing products are again on the rise with the introduction of electronic vapor products^3,4^.

In a 2021 national survey, 13.4% of high school students (2.06 million) reported current use of a tobacco product^5^. The highest percentage of these students reported current use of electronic vapor products (11.3%; 1.72 million), followed by combustible cigarettes (4.4%; 0.66 million), cigars (2.1%; 0.31 million), and cigarettes (1.9%; 0.28 million)^5^. Due to the slowed progress in tobacco control among youth and the associations between youth and adult smoking, tobacco prevention and control among adolescents remain a public health priority^1,2^.

As in adult populations, demographic characteristics are associated with smoking behaviors in high school students^6^. Although rates have narrowed in recent decades, tobacco use remains higher among boys than girls^3,7^. Racial and ethnic differences in smoking patterns have also been observed, with non-Hispanic White youth reporting higher rates of tobacco use than non-Hispanic Black and Hispanic youth (16.2%, 11.0%, and 9.1%, respectively)^5^.

In addition, sexual orientation has emerged as a significant demographic predictor of smoking^8^. Data from several studies indicate that sexual minority youth (SMY) (e.g. lesbian, gay, bisexual, questioning, or unsure) smoke at higher rates than their heterosexual counterparts^4,5,7-9^. Furthermore, SMY start smoking at an earlier age^9^ and are more likely to have friends who smoke^10^, to be exposed to secondhand smoke^11^, and to report higher smoking frequency^9^ and nicotine dependence^8^. SMY are also more likely to transition to regular smoking than heterosexual youth^12^.

The Minority Stress Theory is an established framework for understanding increased risk for poor mental and physical health outcomes among stigmatized minority groups^13^. According to the theory, sexual minorities experience elevated risk due to a combination of general and unique identity-based risk factors. Unique risk factors include sexual identity-specific victimization, harassment, and discrimination. Minority stress is a known risk factor for use of tobacco products among several minority groups, including Black males^14^ and sexual minority women^15^. An Institute of Medicine report on LGBT health stated that additional research is needed to understand the origins of tobacco use disparities among sexual minority populations and to develop interventions to narrow observed differences in health risk behaviors and outcomes^16^. One research area relevant to understanding health risk behaviors among SMY is experience of school-based violence^17^.

School-based violence is an all too commonly reported experience among adolescents^18^. SMY are more likely to report lack of safety in school settings, with more reports of bullying and other forms of victimization^19^. Bullying is a form of repeated and unwanted victimization in which a group or individual desires to intimidate, harm, or exclude a person viewed as vulnerable^20^. Data from national surveillance systems indicate that LGBT and youth unsure of their identity report more bullying on school property than their heterosexual counterparts^20^, and these trends have remained consistent over time^21^. These findings align with those of other regional studies examining school bullying among SMY^22^.

Moreover, electronic bullying or cyberbullying is a subtype of bullying carried out through technology (i.e. blogs, text messages, emails, and social media) against someone not equipped to defend themselves^23^. A systematic review of 27 studies found that 11% to 71% of SMY had experienced this form of victimization^24^. Additionally, in the 2019 Youth Risk Behavior Surveillance System (YRBSS), SMY reported more electronic bullying (27%) than their heterosexual counterparts (13%)^21^.

This study examined associations among sexual minority status, school-based violence, and current tobacco use among youth living in a large urban area. We hypothesized that school-based violence would be associated with current tobacco use behaviors. Furthermore, we hypothesized that higher rates of victimization experiences would explain elevated tobacco use among SMY.

## METHODS

### Study design

A cross-sectional, descriptive, secondary data analysis design was used in this study. The study was classified as exempt by the University of Illinois at Chicago Institutional Review Board, as it involved publicly available, non-identifiable data.

### Data source and sample

Chicago is the third-largest city in the US, is home to a large, racially and ethnically diverse population, and has strong LGBT civil rights protections. However, in Chicago, smoking rates among adults differ significantly by race/ethnicity, community, and sexual orientation, and the city is also plagued by gun-related violence. Chicago is thus an appropriate location for examining differences in tobacco use among diverse youth populations and their associations with school-based violence.

For this study, data for Chicago were obtained from the 2019 YRBSS^25^. The YRBSS is a national school-based survey conducted by school districts every 2 years to assess health-risk behaviors among high school students. A three-stage cluster sample design was used to obtain a nationally representative sample of 9th through 12th-grade students across the US. Sampling weights were applied to adjust for non-responses and oversampling of Black and Hispanic students. In the 2019 YRBSS, 13872 participants responded, for an overall response rate of 60.3%. Additional information about YRBSS methodology, including sampling; survey psychometric properties; response rates; and data collection, processing, weighting, and analysis, has been published elsewhere^25^. The YRBSS dataset for Chicago included an unweighted sample of 1562 participants (weighted population: n=64205).

### Measures

The YRBSS questionnaire contained 99 questions regarding six health-risk behaviors among youth: unintentional injury and violence, tobacco use, alcohol and drug use, sexual behaviors, dietary behaviors, and physical inactivity^25^. Among these, we selected 14 questions related to our study purpose.

*Sexual minority status*

The following YRBSS question was used to determine sexual identity: ‘Which of the following best describes you?’. The response options were ‘Heterosexual (straight)’, ‘Gay or lesbian’, ‘Bisexual’, and ‘Not sure’. Participants who answered ‘Gay or lesbian’, ‘Bisexual’, or ‘Not sure’ were classified as SMY.

*School-based violence*

Five questions in the 2019 YRBSS were used to identify school-based violence status. The first question was: ‘During the past 30 days, on how many days did you not go to school because you felt you would be unsafe at school or on your way to or from school?’ with the response options: 0, 1, 2 or 3, 4–5, and ≥6 days. Responses to this question were included in our study because in previous research, feeling unsafe at school or on the way to or from school have been linked to bullying on school property and cyberbullying among middle and high school students^26-28^.

The following two questions were: ‘During the past 12 months, how many times has someone threatened or injured you with a weapon such as a gun, knife, or club on school property?’ and ‘During the past 12 months, how many times were you in a physical fight on school property?’. The response options for these two questions were: 0, 1, 2 or 3, 4 or 5, 6 or 7, 8 or 9, 10 or 11, and ≥12 times.

The last two questions were: ‘During the past 12 months, have you ever been bullied on school property?’ and ‘During the past 12 months, have you ever been electronically bullied?’. The response options for the last two questions were dichotomous (yes/no). In this examination of school-based violence, it was critical to address bullying on school property and cyberbullying because both types of bullying are linked to students’ social and academic success^29^. Moreover, cyberbullying is a form of relational violence performed by youth and can occur on school property or on the way to or from school^29^.

In this study, school-based violence status was defined as participants responding ‘≥1 day or time’ to any of the first three questions or ‘yes’ to either of the last two questions.

*Current tobacco use*

Current tobacco use was calculated based on four questions^4^: ‘During the past 30 days, how many days did you smoke cigarettes?’; ‘During the past 30 days, how many days did you use an electronic vapor product?’; ‘During the past 30 days, on how many days did you use chewing tobacco, snuff, or dip?’; and ‘During the past 30 days, on how many days did you smoke cigars, cigarillos, or little cigars?’. The response options for each of the four questions were: 0, 1 or 2, 3–5, 6–9, 10–19, 20–29, and 30 days.

Participants who answered ≥1 day^4^ were categorized as current cigarette users, current electronic vapor product users, current smokeless tobacco users, or current cigar users, depending on their responses. Participants who reported any past 30-day use of any product were coded as current tobacco users, and those who said 0 days for all four questions were coded as non-users.

*Covariates*

Three participant demographic characteristics were included as control variables: age (≤15, 16–17, or ≥18 years), sex (female or male), and race/ethnicity (African American, White, Hispanic/Latino, Asian, or Other).

### Statistical analysis

Descriptive statistics were used to describe overall sample characteristics. All variables had less than 5% missing data, and the missing pattern was found to be missing at random. Sample weights and survey strata were employed in the analysis to account for the complex sampling design of the YRBSS^30^. Bivariate analysis (chi-squared test) was used to compare differences in proportions of participants who were current tobacco users and had experienced school-based violence according to their demographic characteristics.

Using generalized structural equation modeling (SEM) with logit link and bootstrapped confidence intervals, path analysis^31^ was performed to estimate the direct and indirect effects of sexual minority status on current tobacco use through school-based violence while controlling for sex, age, and race/ethnicity. A level of 0.05 was considered statistically significant, and 95% confidence intervals for the estimates are reported. All statistical analyses were performed using the complex survey module of Stata version 15 (College Station, TX).

## RESULTS

The participants’ demographic characteristics are given in the Supplementary file Table 1. Most participants (87.85%) identified themselves as belonging to a racial/ethnic minority, with the highest percentage identifying as Hispanic/Latino (49.86%). In addition, 21.11% of participants identified themselves as belonging to a sexual minority. About half of the participants were female (51.2%), and aged 16–17 years (49.4%).

**Table 1:** Prevalence of tobacco product use among Chicago youth, Youth Risk Behavior Surveillance System, Chicago, 2019 (N=1562)

*Characteristics*	*Cigarettes*	*Electronic vapor products*	*Smokeless tobacco*	*Cigars*
	*% (95% CI)*	*% (95% CI)*	*% (95% CI)*	*% (95% CI)*
**Total**	3.86 (2.46–6.01)	12.39 (9.40–16.17)	3.62 (2.20–5.92)	5.54 (3.29–9.19)
**Sex**				
Female	2.81 (1.76–4.45)	11.5 (8.87–14.79)	1.85 (0.94–3.63]	3.76 (2.11–6.63)
Male	4.37 (2.53–7.42)	12.61 (8.98–17.41)	4.4 (2.73–7.01)	6.86 (4.21–10.97)
**Age** (years)				
15	2.46 (1.22–4.92)	8.94 (6.67–11.89)	4.25 (2.09–8.43)	4.19 (2.42–7.17)
16–17	4.12 (2.53–6.63)	13.27 (9.89–17.58)	3.12 (1.82–5.31)	5.51 (3.58–8.40)
18	6.02 (2.51–13.73)	16.32 (8.09–30.18)	3.75 (1.23–10.86)	8.36 (2.11–27.89)
**Race/ethnicity**				
African American	3.11 (1.09–8.52)	11.26 (5.91–20.38)	3.83 (1.33–10.53)	7.23 (2.79–17.49)
White	7.13 (3.42–14.28)	19.25 (12.65–28.18)	1.75 (0.58–5.13)	3.4 (1.72–6.64)
Hispanic/Latino	3.12 (1.87–5.16)	10.71 (7.65–14.79)	2.94 (1.91–4.49)	4.22 (2.56–6.86)
Asian	1.12 (0.14–8.46)	9.63 (4.29–20.21)	0.68 (.09–5.21)	2.33 (.45–11.15)
Other	0	16.53 (6.93–34.50)	0	0
**Gender identity**				
Heterosexual	2.66 (1.78–3.96)	9.54 (7.23–12.48)	1.88 (1.11–3.16)	2.99 (1.89–4.70)
Sexual minority	6.67 (3.31–12.96)	21.59 (15.27–29.61)	8.33 (4.76–14.17)	13.02 (7.30–22.15)

As shown in Table 1, the prevalence of current tobacco use varied by product type. Among the four product types, the highest prevalence of tobacco use was for electronic vapor products (12.39%), followed by cigars (5.54%), cigarettes (3.86%), and smokeless tobacco (3.62%). Electronic vapor products were the most commonly used by heterosexual (9.54%) and sexual minority students (21.59%).

The prevalence of school-based violence is presented in Table 2. Among the five types of violence, not going to school due to feeling unsafe showed the highest prevalence (12.81%), followed by being bullied at school (11.99%) and being electronically bullied (10.98%). Not going to school due to feeling unsafe also showed the highest prevalence in sexual minority students (18.35%). Being bullied at school was the most prevalent violence type for heterosexual students (10.85%).

**Table 2:** Prevalence of school-based violence types among Chicago youth, Youth Risk Behavior Surveillance System, Chicago, 2019 (N=1562)

*Characteristics*	*Did not go to school due to feeling unsafe*	*Threatened/injured with a weapon at school*	*Physical fights at school*	*Bullied at school*	*Electronically bullied*
	*% (95% CI)*	*% (95% CI)*	*% (95% CI)*	*% (95% CI)*	*% (95% CI)*
**Total**	12.81 (9.12–17.70)	9.50 (6.93–12.89)	9.06 (7.17–11.38)	11.99 (10.18–14.08)	10.98 (8.65–13.86)
**Sex**					
Female	12.75 (9.35–17.16)	6.85 (4.29–10.76)	6.47 (4.13–9.99)	12.56 (10.16–15.42)	11.81 (8.99–15.37)
Male	12.22 (7.61–19.05)	11.19 (7.97–15.50)	11.48 (9.32–14.07)	11.56 (8.87–14.95)	9.69 (7.09–13.11)
**Age** (years)					
15	12.97 (9.62–17.28)	11.85 (8.23–16.79)	9.65 (7.44–12.41)	15.64 (12.42–19.51)	15.16 (11.52–19.68)
16–17	12.37 (8.54–17.61)	6.72 (4.83–9.29)	9.21 (6.39–13.11)	9.94 (7.82–12.56)	8.12 (6.14–10.65)
18	13.93 (5.67–30.34)	13.11 (6.54–24.55)	7.50 (3.72–14.56)	10.98 (7.29–16.27)	11.26 (5.59–21.37)
**Race/ethnicity**					
African American	14.04 (6.29–28.42)	12.21 (7.70–18.82)	13.04 (8.51–19.46)	8.90 (5.29–14.57)	8.20 (5.24–12.62)
White	8.06 (4.80–13.23)	6.77 (3.83–11.69)	4.16 (2.04–8.28)	11.62 (7.12–18.42)	11.84 (6.34–21.05)
Hispanic/Latino	13.08 (9.07–18.51)	7.81 (5.35–11.27)	7.96 (6.16–10.23)	13.15 (10.93–15.74)	11.17 (8.34–14.81)
Asian	7.51 (3.68–14.71)	3.54 (1.12–10.63)	4.05 (1.25–12.33)	17.00 (8.62–30.78)	12.82 (6.14–24.86)
Other	17.85 (7.08–38.25)	16.65 (5.10–42.63)	14.00 (5.94–29.54)	18.07 (9.37–31.98)	9.62 (3.24–25.31)
**Gender identity**					
Heterosexual	10.16 (7.15–14.26)	7.31 (5.26–10.09)	7.52 (6.30–8.95)	10.85 (8.86–13.23)	9.69 (7.40–12.59)
Sexual minority	18.35 (12.20–26.68)	15.65 (10.37–22.92)	13.11 (7.68–21.48)	16.19 (11.51–22.3)	14.47 (10.95–18.87)

As presented in Table 3, overall, 16.2% of students reported using a tobacco product in the past 30 days. Sex, age, and race/ethnicity were not significantly associated with current tobacco use. However, sexual minority status was significantly associated with tobacco use, with 30.86% of sexual minority students reporting tobacco use compared to 11.47% of heterosexual students (p<0.001).

**Characteristics Current tobacco use School-based violence % (95% CI):** Prevalence of current tobacco use and school-based violence according to demographic characteristics, Youth Risk Behavior Surveillance System, Chicago, 2019 (N=1562)

*Characteristics*	*Current tobacco use*			*School-based violence*		
	*% (95% CI)*	*χ^2^*	*p*	*% (95% CI)*	*χ^2^*	*p*
**Total**	16.20 (12.35–20.97)			31.76 (26.73–37.26)		
**Sex**		1.47	0.216		0.82	0.524
Female	14.20 (10.91–18.28)			32.41 (27.11–38.20)		
Male	16.69 (12.34–22.19)			30.27 (24.05–37.30)		
**Age** (years)		7.27	0.239		10.28	0.158
15	12.38 (8.90–16.97)			37.11 (32.22–42.27)		
16–17	17.56 (13.69–22.24)			28.81 (23.37–34.94)		
18	19.67 (9.52–36.30)			30.2 (18.78–44.70)		
**Race/ethnicity**		6.26	0.454		5.42	0.579
African American	16.32 (8.85–28.15)			33.45 (23.08–45.71)		
White	20.35 (13.64–29.24)			25.29 (17.75–34.68)		
Hispanic/Latino	13.42 (10.18–17.50)			31.59 (26.01–37.76)		
Asian	10.54 (5.01–20.84)			26.35 (15.88–40.41)		
Other	16.53 (6.93–34.50)			36.75 (20.34–56.92)		
**Sexual identity**		55.91	<0.001		19.48	<0.001
Heterosexual	11.47 (9.08–14.39)			28.1 (23.89–32.74)		
Sexual minority	30.86 (21.74–41.78)			41.15 (32.63–50.24)		

In addition, nearly one-third (31.76%) of students had experienced school-based violence. Sex, age, and race/ethnicity were not significantly associated with school-based violence. However, sexual orientation was significantly related to exposure to violence, with 41.2% of sexual minority students reporting experiencing school-based violence, compared to 28.1% of heterosexual students (p<0.001) (Table 3).

Current tobacco users were more likely to experience school-based violence than non-tobacco users. Current tobacco users reported higher rates of the following experiences than non-tobacco users: did not go to school because they felt unsafe (41.73%; p<0.001), were threatened or injured with a weapon on school property (47.72%; p<0.001), were in a physical fight on school property (37.18; p<0.001), were bullied at school (20.23%; p=0.07), and were electronically bullied (28.66%; p<0.001) (Table 4).

**Table 4:** Prevalence of school-based violence types according to current tobacco use status, Youth Risk Behavior Surveillance System, Chicago, 2019 (N=1562)

*School-based violence type*	*Current tobacco use*	*No tobacco use*	*χ^2^*	*p*
	*% (95% CI)*	*% (95% CI)*		
Did not go to school because they felt unsafe at school or on their way to or from school	41.73 (30.88–53.43)	12.51 (10.15–15.31)	80.62	<0.001
Were threatened or injured with a weapon on school property	47.72 (27.71–68.50)	13.07 (10.74–15.81)	90.25	<0.001
Were in a physical fight on school property	37.18 (25.10–51.1)	12.14 (9.88–14.83)	45.06	<0.001
Were bullied at school	20.23 (13.08–29.95)	13.72 (11.06–16.9)	4.17	0.067
Were electronically bullied	28.66 (17.31–43.54)	12.02 (9.89–14.55)	26.75	<0.001

**Figure 1:**
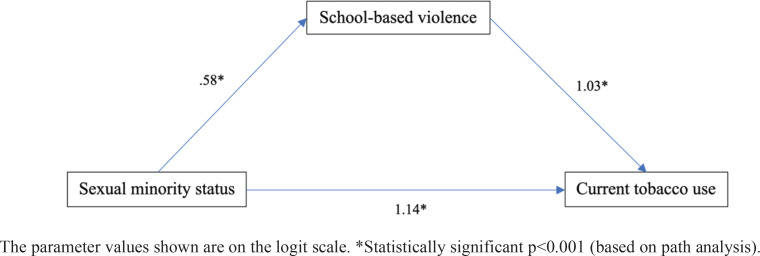
Path analysis model controlling for sex, age, and race/ethnicity, Youth Risk Behavior Surveillance System, Chicago, 2019 (N=1562)

Path analysis was performed to investigate whether sexual minority status was indirectly related to current tobacco use through an association with school-based violence (Figure 1, Table 5). The direct effects observed indicated that both sexual minority status (adjusted odds ratio, AOR=3.13; 95% CI: 1.98–4.96) and school-based violence (AOR=2.79; 95% CI: 1.85–4.21) were strongly related to current tobacco use and each other (AOR=1.78; 95% CI: 1.40–2.59) while controlling for sex, age, and race/ethnicity.

**Table 5:** Path analysis model with logit link and bootstrapped confidence intervals, Youth Risk Behavior Surveillance System, Chicago, 2019 (N=1562)

*Direct effect*	*B*	*SE*	*p*	*AOR*	*95 % CI*
**Current tobacco use**					
School-based violence	1.03	0.20	<0.001	2.79	1.85–4.21
Sexual minority status	1.14	0.22	<0.001	3.13	1.98–4.96
Female	0.25	0.19	0.198	1.28	0.87–1.89
16–17 years	0.80	0.23	0.002	2.23	1.39–3.60
18 years	0.95	0.40	0.027	2.59	1.13–5.95
African American	-0.50	0.35	0.168	0.60	0.29–1.26
Hispanic/Latino	-0.68	0.32	0.043	0.51	0.26–.98
Asian	-0.88	0.55	0.125	0.42	0.13–1.30
Other	-0.62	0.64	0.338	0.54	0.14–2.00
**School-based violence**					
Sexual minority status	0.58	0.12	<0.001	1.78	1.40–2.59
Female	0.18	0.15	0.258	1.19	0.87–1.63
16–17 years	-0.38	0.12	0.005	0.68	0.53–.88
18 years	-0.37	0.26	0.162	0.69	0.41–1.17
African American	0.38	0.35	0.293	1.46	0.71–3.01
Hispanic/Latino	0.27	0.27	0.331	1.31	0.75–2.28
Asian	0.07	0.18	0.698	1.07	0.74–1.55
Other	0.51	0.31	0.115	1.66	0.88–3.14
**Indirect effect**					
Sexual minority status via school-based violence	0.59	0.18	0.001	1.81	1.27–2.57
**Proportion of total effect**					
Sexual minority status via school-based violence	0.34	0.07	<0.001		

The reference group for race/ethnicity is White. The reference group for age is ≤15 years. B: beta regression coefficient. SE: standard error. AOR: adjusted odds ratio.

A significant indirect effect of sexual minority status on current tobacco use was found through the association with school-based violence. This indirect effect increased the odds of current tobacco use by 81% (AOR=1.81; 95% CI: 1.27–2.57), explaining 34.1% of the total association between sexual minority status and current tobacco use; that is, one-third of the association was through the association with school-based violence, while the other two-thirds were attributable to factors not tested in the model.

## DISCUSSION

This study examined rates and predictors of tobacco and other nicotine-containing product use among a diverse sample of high school students in Chicago. Tobacco use was reported by 16% of students, a rate higher than the 13.4% reported by a national sample of high school students^5^. Consistent with national trends^5,7^, use of electronic vapor products was most commonly reported by Chicago youth, followed by cigars and cigarettes.

The highest rate of overall tobacco use was among White youth, followed by African American, and Hispanic/Latino youth. However, variations were seen in the types of products used. A higher percentage of White students reported use of electronic vapor products and cigarettes, while a higher percentage of Black youth reported use of cigars and smokeless tobacco.

According to the bivariate analysis, sexual orientation was the strongest demographic correlate with tobacco use. Depending on the product involved, rates of tobacco use among SMY were between 2.3 and 4.4 times higher than for their heterosexual counterparts. These findings are consistent with a large and growing body of literature documenting significant inequalities in tobacco use based on sexual orientation^4,5,7-9^.

Examining the mechanisms associated with elevated health risk behaviors among marginalized social groups is vital for developing prevention and intervention programs. We hypothesized that victimization experiences would be directly associated with tobacco use behaviors, a hypothesis that our findings support. In our study, nearly one-third of youth reported experiencing at least one form of school-based violence, with a higher rate (41.2%) reported by SMY than by heterosexual youth. Path analysis confirmed that school-based violence was directly associated with increased likelihood of current tobacco use while controlling for sex, age, and race/ethnicity. These findings are consistent with previous research in which youth with in-person and electronic bullying experience were at greater risk for several risk behaviors, including tobacco use^32^. Among youth and adults alike, exposure to violence is a known risk factor for adverse mental health outcomes^33^. Furthermore, exposure to violence is strongly associated with engagement in health-compromising behaviors such as smoking and other substance use^32^.

Research findings suggest that individuals exposed to violence and other traumatic events use substances to regulate negative affect such as anxiety^17^. Based on this research, we hypothesized that the relationship between sexual minority status and tobacco use would be mediated by greater victimization experience. This hypothesis was also supported. We found that the effect of sexual minority status on self-reported smoking status was mediated by exposure to school-based violence. That is, sexual minority status increased the likelihood of experience of school-based violence, and such experience influenced smoking status. These findings align with the literature linking sexual minority status to increased risk for multiple forms of victimization^17,34^ and associating victimization experiences with increased risk for smoking^32,34,35^. For example, a study of young adults reported that lesbians/gays who were in physical fights or were physically assaulted showed higher probabilities of being current smokers than lesbians/gays without victimization experiences^34^.

### Implications

Public health policy is the most effective strategy for reducing tobacco use among youth and adults^36^. Chicago and the State of Illinois have passed a series of legislative measures that have influenced smoking rates among youth. These measures included raising the legal age to purchase tobacco products from 18 to 21 years, requiring stores to post warning signs disclosing that sales to underage individuals are prohibited, restricting the sale of tobacco products by clerks aged <21 years, banning tobacco discounts and sampling, and increasing taxes on tobacco products^37^. These actions have resulted in reduced tobacco use among teens over time^37^. However, additional efforts are needed to reduce youth access to electronic nicotine delivery systems (ENDS).

In 2020, the City of Chicago passed a vaping ban that prohibited the sale of all flavored nicotine-containing vaping liquids^38^. Banning of flavored vaping products, age restrictions, and stepped-up enforcement efforts should reduce access to and use of ENDS among Chicago youth^38^. However, more specific actions are warranted to address the disproportionate tobacco use among SMY compared to their heterosexual counterparts.

Violence is a primary risk factor for SMY, and school-based policies and interventions are essential for protecting the physical and emotional health of these youth. Although several school systems have attempted to implement anti-victimization programs, many of these programs did not specifically address LGBT youth^39^. Alternatively, student-led organizations may offer a promising approach for protecting SMY from victimization. For example, gay-straight alliances (GSA) are student-led clubs that provide a safe space for socialization of LGBT students and their allies. A study provided strong evidence of the association between the presence of GSAs in schools and lower levels of self-reported victimization among students, including homophobic victimization, fear for safety, and hearing homophobic remarks^40^. Also, GSAs are a cost-effective strategy, as they require minimal resources and no formal curriculum^40^.

Smoking prevention campaigns and interventions targeted to SMYs are also needed. A scoping review identified several community-based, group counseling cessation interventions that have been implemented for LGBT smokers across the US with favorable results^41^. For example, three programs based on the American Lung Association’s Freedom from Smoking Program – *Put it Out, Bitch to Quit, and Call It Quits* – were culturally tailored to LGBT individuals in Chicago and demonstrated a quit rate of 32% at 1 month post-quit date across the three programs^41^. However, none of the studies included in the scoping review specifically targeted LGBT youth, and the findings from studies focusing on LGBT adults cannot be assumed to be relevant to SMY.

Additionally, the Network for LGBT Health Equity released a report entitled *MPOWERED: Best and promising practices for LGBT tobacco prevention and control* ^42^ that provided a roadmap for eliminating LGBT tobacco use disparities. Eight best and promising practices included: 1) monitoring the tobacco epidemic; 2) protecting from secondhand smoke; 3) offering support to quit; 4) warning of the impact of tobacco use; 5) enforcing protections; 6) raising tobacco taxes; 7) evaluating programs and disseminating findings; and 8) diversifying the tobacco control movement^42^. Given the limited evidence available specifically for LGBT youth, health professionals and policymakers may need to draw upon parallel evidence from other LGBT populations or settings, to facilitate adoption of smoking prevention and cessation programs for SMY.

### Strengths and limitations

The YRBSS has numerous strengths, including its large probability sample, the racial/ethnic diversity of the sample, and its inclusion of a range of risk and protective factors related to health risk behaviors. Given these strengths, the YRBSS has been used extensively to examine trends in tobacco use among high school students. Furthermore, the YRBSS asks about sexual orientation, which has emerged as an essential risk factor for mental and physical health among adolescents.

Despite the strengths of this survey, some limitations should be noted. First, the YRBSS is cross-sectional in nature, precluding identification of causal relationships. In addition, tobacco use is self-reported by survey respondents, having an unknown influence on resulting tobacco use estimates. Also, the YRBSS does not include a standardized measure of gender identity, preventing examination of the impact of this established risk factor on tobacco use. Additional research is needed to both replicate and extend the findings of this study to be inclusive of all sexual and gender minority youth.

## CONCLUSIONS

Using Chicago-specific data from the national 2019 YRBSS, this study revealed school-based violence and tobacco use inequities between SMY and their heterosexual peers. Study findings point to key issues that must be addressed, including the high rates of tobacco use among SMY and the role of school-based violence in increasing tobacco use in this minority population. Our findings provide greater understanding of the factors and mechanisms associated with elevated risks of tobacco use in SMY, an understanding that can inform development of appropriate interventions for this marginalized social group. The results highlight the need for prevention and cessation efforts for tobacco use among SMY as well as school-based interventions to reduce their exposure to violence. Moreover, implementation of comprehensive public health policy and approaches at the national and local levels is essential to reduce tobacco use disparities among SMY.

## Data Availability

The data supporting this research is available from the following sources: https://www.cdc.gov/healthyyouth/data/yrbs/index.htm
