# Supplementary material for: Sexual minority status, school-based violence, and current tobacco use among youth
Source: Tob Prev Cessat. 2022 Dec 15;8:46. doi: 10.18332/tpc/156110 (PMC9753573; doi:10.18332/tpc/156110)
Supplement: Supplementary file 1 [file TPC-8-46-s1.pdf]

Supplementary Table 1. Demographic characteristics of participants, Youth Risk Behavior Surveillance System, Chicago, 2019 (N = 1,562)

Characteristic	Unweighted Frequency	Weighted percentage	95% CI
**Sex**			
Female	769	51.22	47.13-55.29
Male	769	48.78	44.71-52.87
**Age group (years)**			
≤ 15	500	33.77	25.85-42.73
16 - 17	834	49.44	42.31-56.58
≥ 18	224	16.79	12.69-21.87
**Race/Ethnicity**			
African American	278	30.47	21.96-40.55
White	181	12.15	9.09-16.04
Hispanic/Latino	905	49.86	41.63-58.10
Asian	95	5.48	3.71-8.03
Other	45	2.04	1.47-2.83
**Sexual identity**			
Heterosexual	1,167	78.89	75.27-82.10
Gay or lesbian	48	3.85	2.42-6.07
Bisexual	169	11.78	9.38-14.70
Not sure	83	5.48	4.36-6.86

CI: confidence interval.

© 2022 Duangchan C. et al.
